# Signal Processing Circuits and Systems for Smart Sensing Applications

**DOI:** 10.3390/s23125492

**Published:** 2023-06-10

**Authors:** Norbert Herencsar, Khaled N. Salama

**Affiliations:** 1 Department of Telecommunications, Brno University of Technology, Technicka 3082/12, 616 00 Brno, Czech Republic; 2 Computer, Electrical and Mathematical Sciences and Engineering Division, King Abdullah University of Science and Technology, Thuwal 23955, Saudi Arabia; khaled.salama@kaust.edu.sa

The rising demand for reliable, real-time, low-maintenance, cost-efficient monitoring systems with a high accuracy is becoming increasingly more notable in everyday life. One of the main driving forces behind this is the Internet of Things. In this regard, the growing interest in analog and mixed signal integrated signal processing circuits in portable and wearable smart-sensor-related technologies and interfaces used for healthcare and sports performance monitoring, environmental monitoring, and agriculture, food control, or energy conservation purposes is an important benchmark to measure this demand. This demand also requires eco-friendly integrated circuits and devices with voltage and power management. On top of that, other characteristics such as quietness, small size, high signal-to-noise ratios, and so on should be standard sensor circuit features. All these design concerns present serious design challenges to researchers in this emerging field.

This Special Issue is aimed at highlighting the advances in the development, design, implementation, modeling, and validation of integrated circuits and systems in smart sensing applications for the benefit of humanity. The guest editors are pleased to introduce a collection of the five best articles focusing on this specially selected topic. In the following, brief descriptions of the accepted articles are presented.

The global coronavirus disease pandemic (COVID-19) has dramatically changed human lives and resulted in the limitation of physical activities, which has led to various health issues such as cardiovascular diseases, diabetes, and gout. Physical activity is a double-edged sword, providing significant health benefits but causing irreparable damage due to, e.g., falls, leading to fatal and non-fatal injuries. Continuous monitoring is crucial during quarantine to detect falls. Khan M.B. et al. developed a non-contact smart sensing platform using software-defined radio technology to monitor physical activities during quarantine [1]. The platform is intelligent, portable, and multi-functional. Falls are classified with a 99.7% accuracy using a fine tree algorithm. This smart sensor opens new research directions to detect COVID-19 symptoms and monitor non-communicable and communicable diseases.

Measuring the weather in urban environments is crucial for smart cities to prevent flooding and stop emergency services being blocked during heavy rain. A dense measurement network of low-cost rain gauges is required for this purpose Clemens C. et al. presented an inductive rain gauge based on the eddy current principle [2]. The gauge consists of a coil under a metal plate, which oscillates when hit by raindrops and changes the frequency of the resonant circuit. The sensor is cost-effective, energy self-sufficient, and transmits data wirelessly via LoRaWAN, enabling flexible use and a wide coverage. Preliminary experimental investigations demonstrate that the size of rain droplets can be detected and categorized based on frequency changes. This measurement principle provides high-resolution site-specific data that can be used to calculate precipitation in urban areas.

Impedance spectroscopy has become an essential non-invasive tool for quality assessment measurements of the biochemical and biophysical changes of plant tissues. The electrical behavior of biological tissues can be captured by fitting its bio-impedance data to a suitable circuit model. Gadallah S.I. et al. investigated the use of power-law filters in the circuit modeling of bio-impedance [3]. Using a meta-heuristic optimization method, the proposed models are fitted to the experimental data of eight different fruit types. Impedance measurements are obtained using a Biologic SP150 electrochemical station, and the percentage error between the actual impedance and the fitted model impedance is reported. It is found that a circuit model consisting of a combination of two second-order power law low-pass filters shows the lowest fitting error.

In article [4], Cheon S.-I. et al. proposed an error-tolerant and power-efficient impedance measurement scheme for bioimpedance acquisition. Unlike other architectures, the proposed architecture measures both the magnitude and real part of the target impedance, obtaining phase information using the ratio between them. A reference resistor compensates for errors caused by delay, and an additional magnitude measurement path cancels system nonlinearity and enhances the settling speed by ratio-based detection. Compared to conventional detection, the proposed circuit improved the accuracy by 30% and settling time by 87.7%. The integrated circuit consumes only 513 μW for a wide frequency range of 10 Hz to 1 MHz, with maximum errors of 0.3% and 2.1° for the magnitude and phase, respectively.

Electronic textiles (e-textiles) and wearable computing have progressively emerged in the last decade. Since market interest and predictions have grown, the research into increasing the reliability and durability of wearables and e-textiles is developing rapidly. The washability of different integrated devices and their resistance to mechanical stress are the main obstacles being tackled. In [5], Veske P. et al. focused on developing and improving the thermoplastic polyurethane (TPU) lamination integration method for e-textiles. A stretchable copper–polyimide-based circuit was laminated onto a knitted fabric using various TPU films and stacks. The study shares measurable characteristics to determine which material assembly and design ensures the highest durability for the electronics part without losing its original textile softness, flexibility, and stretchability.

In conclusion, this Special Issue contains a series of excellent research works on signal processing circuits and systems for smart sensing applications. This collection of five articles is highly recommended and believed to be interesting, inspiring, and motivating for researchers and other professionals.
